# Association Between Pre-Transplant Oral Health and Post-Liver Transplant Complications

**DOI:** 10.3389/ti.2023.11534

**Published:** 2023-09-12

**Authors:** Annika Emilia Olander, Jaana Helenius-Hietala, Arno Nordin, Johanna Savikko, Hellevi Ruokonen, Fredrik Åberg

**Affiliations:** ^1^ Department of Oral and Maxillofacial Diseases, Helsinki University Hospital and University of Helsinki, Helsinki, Finland; ^2^ Department of Transplantation and Liver Surgery, Helsinki University Hospital and University of Helsinki, Helsinki, Finland

**Keywords:** oral health, liver transplantation, acute rejection, infection foci, oral disease

## Abstract

Oral disease is linked with systemic inflammation and various systemic conditions, including chronic liver disease. Liver transplantation (LT) candidates often need dental infection focus eradication, and after LT, there is high risk of many inflammation-related complications. We studied whether pre-LT dental status is associated with the occurrence of post-LT complications. This study included 225 adult LT recipients whose teeth were examined and treated before LT, and 40 adult LT recipients who did not have pre-LT dental data available. Data on post-LT complications were collected from the national liver transplant registry and followed up until the end of July 2020. Worse pre-LT dental status was associated with a higher risk of acute rejection post-LT compared to patients with good dental status. Worse dental status was also associated with higher 1-year-post-LT ALT levels and lower albumin levels. In conclusion, poor pre-LT oral health seems to associate with an increased risk of post-LT acute rejection and with elevated ALT levels and decreased albumin levels, suggesting an effect on post-LT liver health. Therefore, prevention and treatment of oral and dental diseases should be promoted early in the course of liver disease.

## Graphical Abstract

**Figure:**
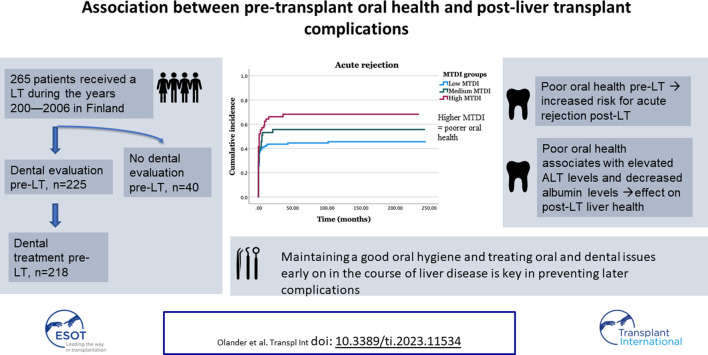

## Introduction

A relationship between poor oral health and liver disease has been presented previously. The systemic inflammation caused by oral infections seems to accelerate the progression of chronic liver disease (CLD) [1–3], and CLD, in turn, seems to affect oral diseases [4, 5]. Oral infection foci need to be eliminated before liver transplantation (LT) to avoid severe systemic complications [6]. A significant link between a lack of pre-LT dental treatment and post-LT systemic infections has been suggested in patients with acute liver failure (ALF) [7]. In a study by Göbel et al. [8], dental infection foci was associated with a higher risk of pre- and post-LT bacterial infections. After transplantation, patients use immunosuppressants for the rest of their lives. During the first year post-LT in particular, but also later, patients are prone to severe, even fatal, infections [9, 10].

The need for dental and periodontal treatment has proved to be high among LT recipients, and poor oral hygiene [4, 11, 12]. Compared to healthy controls, LT candidates are shown to have a higher prevalence of apical periodontitis and other oral diseases (i.e., dental caries, periodontal and oral mucosal diseases) [13, 14]. Furthermore, the presence of oral diseases has been linked to a higher risk of mortality in liver cirrhosis patients [15, 16]. Although the connection between oral health and liver disease is increasingly being studied, studies about the connection between oral infection foci and post-LT complications remain scarce.

Common complications after LT include graft rejection, biliary strictures, and various infections [17]. LT recipients also have increased risks of many diseases, such as diabetes, hypertension, cardiovascular disease, and cancer [17]. The risk factors for these complications in LT recipients are only partly known.

The objective of this study was to examine the connection and impact of pre-LT oral health on post-LT complications, such as graft rejection, cardiovascular disease, infections, cancer, and mortality. We hypothesized that LT recipients with worse oral health have more post-LT complications.

## Patients and Methods

This study was performed in accordance with the Declaration of Helsinki and has been approved by the HUS ethics committee (192/13/03/02/2008. 12 August 2008).

This study included all 265 adult LT recipients who received a liver graft during the years 2000–2006 at Helsinki University Hospital (HUS), which is the only LT center in Finland. Children under 15 years were excluded from this study. Of these patients, 225 (total 233 liver transplantations) underwent a dental evaluation prior to transplantation. Forty patients either lacked sufficient dental data or did not undergo a pre-LT dental evaluation. A flowchart of the patients included in the study is shown in Supplementary Figure S1. The underlying indications for transplantation are presented in Supplementary Table S1.

### Complication Data and Laboratory Values

Outcome data until the end of July 2020 were obtained from the national LT registry. The national LT registry contains follow-up data on relevant complications following LT. These data are collected during annual follow-up visits at the transplantation center and local hospitals [10]. Complications were grouped into severe infections requiring hospital care, cardiovascular disease, cancer, acute or chronic rejection, death or re-transplantation, and incidental diabetes, and were considered outcomes. For the 225 patients with dental data available, we also registered the model for end-stage liver disease (MELD) scores at transplantation, and 1 year-post-LT laboratory values for alanine aminotransferase (ALT), alkaline phosphatase (ALP), albumin, total bilirubin, gamma-glutamyl transferase (GGT), and c-reactive protein (CRP).

### Dental Examination and Parameters

Dental screening for infection foci was performed for 225 patients before transplantation, and 191 of these patients underwent a clinical and radiographic (panoramic tomography x-ray) dental examination. However, 32 patients were evaluated only based on the panoramic tomography x-ray and two did not undergo either a full clinical examination or panoramic tomography x-ray because they were edentulous. Furthermore, for 218 of the 225 patients, acute infection foci were eliminated prior to transplantation. The clinical examination consisted of examining the extraoral status, the oral mucosa, the dentition and occlusion, and the periodontium. The oral health of the patients was evaluated using the modified total dental index (MTDI) score [18]. The MTDI score, presented in Table 1, considers the amount of caries lesions, edentulous jaws, radiological alveolar bone loss, number of apical lesions, and pericoronitis. A total score was counted for each patient, with a maximum possible score of 10 points. The patients were further divided into equal tertiles based on their MTDI score: low MTDI (0–2), medium MTDI [3] and high MTDI [4–10]. The low MTDI group was considered to have a low number of dental infection foci, whilst the high MTDI group was considered to have a high number of dental infection foci. A DMFT (decayed, missing, filled teeth) score was also registered for the patients, along with the number of extracted teeth.

**TABLE 1:** Definition and scoring of the Modified Total Dental Index (MTDI).

	Points
Caries	
No caries lesions	0
1–3 caries lesions	1
4–7 caries lesions or no teeth in mandible or maxilla	2
≥8 caries lesions or radix or no teeth	3
Periodontitis	
No alveolar bone loss	0
Alveolar bone loss in cervical third	1
Alveolar bone loss in middle third	2
Alveolar bone loss in apical third	3
Periapical lesions	
1 periapical lesion or vertical bone pocket or both	1
2 periapical lesions	2
≥3 periapical lesions	3
Pericoronitis	
Absent	0
Present	1
Maximum score	10

### Data Analyses

The data analyses were performed using IBM SPSS Statistics software version 27.0 (SPSS, Inc., Armonk, NY, United States). Data are given as mean ± SD or count (%). Continuous variables were assessed for normality using the Shapiro–Wilk test and histograms. None of the continuous variables were normally distributed. Differences between groups were analyzed using the Fisher–Freeman–Halton exact test and, for nonparametric variables, using the Kruskal–Wallis test. For *post hoc* test Z-test for proportions and Dunn’s test, both Bonferroni corrected, were used as appropriate. The association between MTDI score and post-LT complications were first analyzed using univariate Cox regression analysis. Variables that were found to be significant in univariate analyses were further analyzed in multivariate analyses. Dental parameters and complications were adjusted for age, sex, indication for transplantation, smoking, pre-LT diabetes, MELD score at LT, and the number of LTs. Laboratory values were adjusted for sex, age at LT and MELD score at LT. We also analyzed whether there was a difference in the occurrence of complications between patients with or without pre-LT dental data with Cox regression models. Hazard ratios (HRs) with 95% confidence intervals (CIs) are reported. Linear regression analysis was used to assess the association between MTDI score and laboratory values, and *p*-values <0.05 were considered statistically significant.

## Results

### Basic Characteristics

Basic characteristics for patients with low, medium, and high MTDI scores are presented in Table 2. Patients in the high MTDI group were older compared to the low MTDI group (*p* = 0.027). The high MTDI group had a larger proportion of patients with acute rejection post-LT than the low MTDI group (*p* = 0.018). Analyses on dental parameters showed that patients in the low MTDI group had lower DMFT scores than patients in the medium MTDI group (*p* = 0.037) and the high MTDI group (*p* = 0.008). Patients in the low MTDI group also had more teeth before dental treatment compared to patients in the medium MTDI group (*p* = 0.005). Furthermore, patients in the high MTDI group had more teeth extracted pre-LT compared to the low MTDI group (*p* < 0.001). Moreover, patients in the low MTDI group had higher 1 year-post-LT albumin levels compared to patients in the medium MTDI group (*p* = 0.013) and the high MTDI group (*p* = 0.008).

**TABLE 2:** Basic characteristics of the 218 LT patients who underwent elimination of dental infection foci pre-LT and the underlying indication for transplantation.

Parameter	Low MTDI	Medium MTDI	High MTDI	*p*-value
Number of patients	116	42	60	
Age^a^ (years)	46.5 (±13.1)	50.9 (±11.2)	52.2 (±9.2)	**0.011** ^b^
Sex^c^ (male/female)	58 (50%)/58 (50%)	25 (60%)/17 (40%)	40 (67%)/20 (33%)	0.101
Indication for transplantation^c^				
Chronic liver disease	92 (79%)	34 (81%)	47 (78%)	
I) Primary sclerosing cholangitis	30 (26%)	9 (21%)	10 (17%)	
II) Primary biliary cholangitis	15 (13%)	6 (14%)	9 (15%)	
III) Alcohol cirrhosis	15 (13%)	9 (21%)	17 (28%)	
IV) Cryptogenic cirrhosis/NASH	12 (10%)	4 (10%)	7 (12%)	
V) Other cirrhosis	12 (10%)	8 (19.%)	7 (12%)	
VI) Other CLD^d^	16 (14%)	3 (7%)	2 (3%)	
Acute liver failure	12 (10%)	3 (7%)	6 (10%)	
Tumor (all)^e^	11 (10%)	5 (12%)	7 (12%)	
I) Tumor (no other CLD)^f^	4 (3%)	0 (0%)	2 (3%)	
Metabolic disease	1 (1%)	0 (0%)	0 (0%)	
Complication data^c^				
No of patients: ≥1 complication	115 (99%)	42 (100%)	60 (100%)	1.000
No. of patients: no complications	1 (1%)	0 (0%)	0 (0%)	
No. of patients: Survival				0.133
I) Retransplantation	12 (10%)	5 (12%)	2 (3%)	
II) Death	31 (27%)	13 (31%)	26 (43%)	
No. of patients: Infection	77 (66%)	31 (74%)	39 (65%)	0.601
No. of patients: cardiovascular disease	11 (10%)	7 (17%)	11 (18%)	0.186
No. of patients: Incident diabetes	33 (28%)	14 (33%)	14 (23%)	0.539
No. of patients: Hypertension	66 (57%)	24 (57%)	37 (62%)	0.823
No. of patients: Cancer	31 (27%)	10 (24%)	18 (30%)	0.878
No. of patients: Acute rejection	52 (45%)	23 (55%)	40 (67%)	**0.023** ^g^
No. of patients: Chronic rejection	3 (3%)	0 (0%)	1 (2%)	0.816
Dental parameters^a^				
DMFT score	20.6 (±9.2)	24.6 (±7.3)	25.5 (±4.8)	**0.003^h^**
Number of teeth pre dental treatment	25.1 (±6.6)	18.3 (±11.9)	23.5 (±6.5)	**0.003^i^**
Number of extracted teeth pre-LT	1.4 (±2.2)	2.4 (±3.5)	7.4 (±5.1)	**<0.001^l^**
MELD score at LT^a^	18.4 (±8.6)	19.4 (±7.9)	18.0 (±7.3)	0.712
Laboratory values at 1 year post-LT^a^				
P-ALT (U/L)	32 (±23)	34 (±33)	55 (±59)	0.102
P-ALP (U/L)	144 (±113)	163 (±143)	155 (±101)	0.415
*P-Bilirubin* (µmol/L)	15 (±8)	14 (±7)	15 (±13)	0.386
P-Albumin (g/L)	38 (±4)	36 (±4)	36 (±4)	**0.001^k^**
P-GGT (U/L)	89 (±188.03)	88 (±140)	131 (±212)	0.117
P-CRP (mg/L)	3 (±14)	3 (±10)	4 (±15)	0.582

Patients with low (0–2), medium (3) and high (4–10) MTDI scores are compared.

Abbreviations: ALP, alkaline phosphatase; ALT, alanine aminotransferase; CLD, chronic liver disease, CRP, c-reactive protein; DMFT, decayed, missing, filled teeth; GGT, gamma-glutamyl transferase; LT, liver transplantation; MELD, model of end-stage liver disease; MTDI, modified total dental index; NASH, non-alcoholic steatohepatitis. Non-parametric variables were analyzed using Kruskal-Wallis test, and categorical variables using Fisher’s exact test. Post-hoc testing was done using, as appropriate, Dunn’s test and the Z-test for proportions, respectively, with both being Bonferroni corrected.

^a^ Data given as mean (SD).

^b^ In pairwise comparison, patients in the high MTDI group were significantly older than in the low MTDI group (*p* = 0.027).

^c^ Data given as *n* (%).

^d^ Other chronic liver diseases includes Budd–Chiari disease, polycystic disease, extrahepatic biliary atresia, congenital biliary fibrosis, alpha-1 antitrypsin deficiency, choledochal cyst, Caroli disease, and cystic fibrosis.

^e^ All patients with a tumor as the underlaying cause.

^f^ Patients with a tumor as the only underlaying cause.

^g^ The high MDTI group had a significantly larger proportion of patients with acute rejection compared to the low MTDI group (*p* = 0.018).

^h^ In pairwise comparison, patients in the low MTDI, group had significantly lower DMFT scores compared to patients in the medium MTDI group (*p* = 0.037) and the high MTDI group (*p* = 0.008).

^i^ In pairwise comparison, patients in the low MTDI group had significantly more teeth before dental treatment compared to patients in the medium MTDI group (*p* = 0.005).

^j^ In pairwise comparison, patients in the high MTDI group had significantly more teeth extracted compared to patients in the low MTDI group (*p* < 0.001) and medium MTDI, group (*p* < 0.001).

^k^ In pairwise comparison, patients in the medium MTDI group (*p* = 0.013) and high MTDI, group (*p* = 0.008) had significantly lower albumin values compared to patients in the low MTDI group.

Basic characteristics, including background, dental and outcome parameters, for patients with and without dental data are presented in Supplementary Table S1. The ratio between men and women differed significantly (*p* < 0.001) between the two groups; otherwise, no significant differences were observed.

### Association Between Pre-LT MTDI Score and Post-LT Complications

**FIGURE 1:**
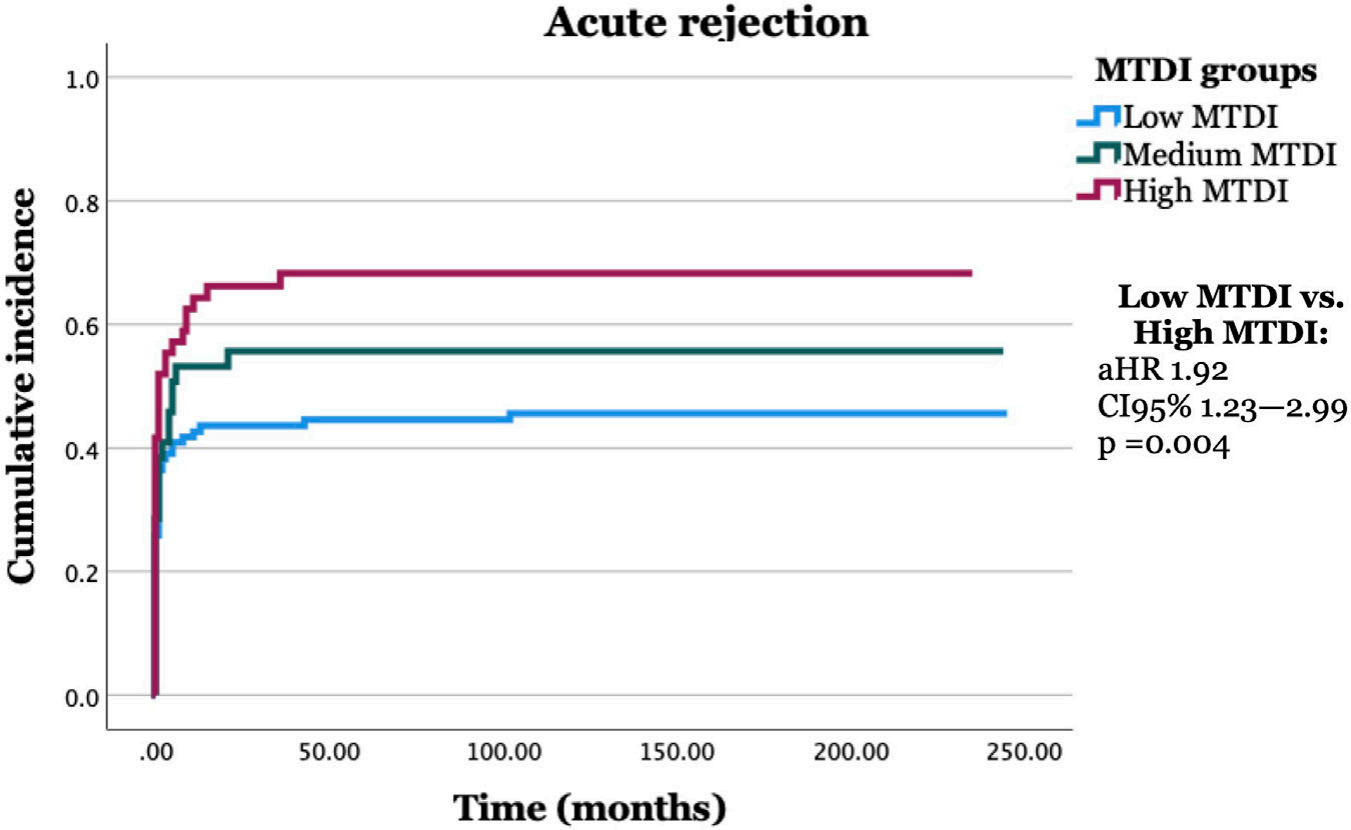
Liver transplant (LT) recipients with a high need for dental treatment (high MTDI) and multiple infection foci pre-LT expressed significantly more acute rejection post-LT compared to LT recipients with a low or no need for dental treatment (low MTDI) pre-LT.

The correlation between pre-LT MTDI score and post-LT complications was analyzed with a Cox regression. The results are shown in Supplementary Table S2. In univariate analysis with MTDI group (low, medium, high) as the independent variable, patients with high MTDI had a significantly higher risk of acute rejection compared to patients in the low MTDI group (HR 1.7, CI 95% 1.1–2.6, *p* = 0.012). No other significant findings between MTDI score and other complications were seen. When investigating the correlation between MTDI score and acute rejection further, we found that the results also remained significant after adjusting for age, sex, smoking, indication for LT, pre-LT diabetes, MELD score at transplantation, and re-transplant status (HR 1.9, CI 95% 1.2–3.0, *p* = 0.004). Furthermore, when adjusting for the time between dental evaluation and LT or the interaction variable between MTDI groups and the time between dental evaluation and LT, the results remained significant. Results are presented in Figure 1.

Moreover, the proportion of LT recipients with more than one acute rejection episode was significantly higher (*p* = 0.014) in the high MTDI group (20%) compared to the low MTDI group (6%). No significant difference was found when analyzing the correlation between MTDI group and early (<3 months post-LT) or late (>3 months post-LT) acute rejection.

### Association of Pre-LT MTDI Score and Dental Data With 1 year-post-LT Laboratory Values

In Table 3, the results of the linear regression analysis of the correlation between MTDI score as a continuous variable and 1 year-post-LT laboratory values for ALT, ALP, bilirubin, albumin, GGT and CRP are shown. There was a significant association between a higher MTDI score and higher ALT values (*β* = 4.0, CI 95% 1.2–6.7, *R* ^2^ = 0.039, *p* = 0.005) and with lower albumin values (*β* = −0.5, CI 95% −0.8 to −0.2, *R* ^2^ = 0.052, *p* = 0.001). The results remained significant after adjusting for sex, age at LT, and MELD score at LT (*β* = 4.2, CI 95% 1.3–7.1, *R* ^2^ = 0.04, *p* = 0.005 and *β* = −0.5, CI 95% −0.8–0.2 *R* ^2^ = 0.1, *p* = 0.002, respectively).

**TABLE 3:** Univariate linear regression analysis of the connection between MTDI score and laboratory values 1 year post-transplantation.

Laboratory value	*β*	CI 95% for B	*R* ^2^	*p*-value
P-ALT (*U/L*)	3.97	1.20–6.74	0.038	**0.005**
P-ALP (*U/L*)	3.44	−4.87–11.75		0.415
P-Bilirubin (µmol/L)	0.09	−0.58–0.76		0.782
P-Albumin (*g/L*)	−0.48	−0.77 to −0.19	0.052	**0.001**
P-GGT (*U/L*)	12.44	−0.93–25.82		0.068
P-CRP (*mg/L*)	0.78	−0.20–1.75		0.119

Abbreviations: ALP, alkaline phosphatase; ALT, alanine aminotransferase, CRP, c-reactive protein, GGT, gamma-glutamyl transferase.

*β* = regression coefficient.

These results concern 218 patients whose teeth were examined and treated pre-transplantation.

MTDI score (0–10) analyzed as a continuous variable.

When further analyzing whether the pre-LT number of teeth correlates with 1 year-post-LT ALT and albumin values in separate linear regression analysis, we found that fewer teeth associated with lower albumin values (*β* = 0.2, CI 95% 0.04–0.2, *R* ^2^ = 0.05, *p* = 0.002).

## Discussion

The main finding in our study was that patients with high pre-LT MTDI scores, indicating a worse dental status, seemed to have a higher risk of acute post-LT rejection compared to patients with low pre-LT MTDI scores. Furthermore, a higher MTDI score seems to associate with higher 1 year-post-LT ALT levels and lower albumin levels.

To the best of the authors’ knowledge, the association between poor dental status and acute rejection post-LT has not been reported in LT recipients previously. In our study, we found that a higher MTDI score (worse oral health) independently predicts acute rejection. The correlation remained significant even when adjusted for confounders. Previous studies on kidney transplant recipients show contradictory results on the matter. Zweich et al. [19] showed that poor oral hygiene was an indicator for increased risk of hospitalization and acute rejection, and found that the Community Periodontal Index of Treatment Needs correlated with acute rejections in kidney transplant recipients. However, other studies have demonstrated no correlation between pre-transplant oral health and graft rejection [20, 21]. One study showed that severe periodontitis in kidney transplant recipients was independently associated with a lower incidence of acute T-cell-mediated rejection, which was hypothesized to depend on the immunomodulatory effect of periodontitis [22].

In this study, all acute odontogenic infection foci were treated pre-LT. However, not all patients underwent periodontal treatment systematically. A potential link between periodontitis/gingivitis and graft rejection could be a periodontitis-related, IL-6-modulated, pro-inflammatory state. IL-6 production can be induced by both pathogen-associated molecular patterns and pro-inflammatory cytokines, and it seems to have a pro-inflammatory effect on the adaptive immune response [23]. In the solid organ transplantation context, IL-6 has been shown to promote acute allograft rejection [24–26]. Periodontitis has been shown to increase levels of proinflammatory cytokines such as TNF-alfa, IL-1 and IL-6 [27]. In particular, patients with chronic periodontitis have elevated levels of proinflammatory cytokines, including IL-6, in the gingival crevicular fluid and serum compared to healthy controls [28, 29]. Periodontal treatment, in turn, is shown to lower the levels of these proinflammatory cytokines [29, 30]. Thus, we hypothesize that untreated periodontal inflammation plays a role in the development of acute rejection.

Previous publications on the association between liver enzymes and oral health are contradictory and are mainly focused on periodontitis. Some studies show no association between liver enzyme values and alveolar bone loss in patients without liver disease [31, 32], while other studies show higher liver enzyme levels in patients with periodontitis [33, 34]. Some studies hypothesize that periodontal disease might be a risk factor for the development of non-alcoholic steatohepatitis [35–37] and chronic liver disease [38]. Our results showed significantly higher ALT levels and significantly lower albumin levels in patients with worse dental health. ALT is a highly liver-specific enzyme, and it being elevated in patients with worse oral health supports our hypothesis that untreated oral or dental inflammation might also influence the liver post-LT. Poor nutrition is shown to reduce albumin production [39]. In our study, worse oral health and fewer teeth were associated with lower albumin values. Hence, worse oral health and fewer teeth may contribute to malnutrition by negatively affecting chewing capacity and mastication; this connection has previously been discussed in patients with chronic kidney disease [40].

Our study reports novel findings on the connection between oral health and post-LT complications. Strengths of our study include the long follow-up time, the utilization of data from the national LT registry, and using both clinical and laboratory data. Despite the novel findings, our study has limitations. In this study setting we could not collect detailed data on the patients’ periodontal status and periodontal treatment. Furthermore, our study analyzed oral health as a whole, and we were not able to exam different oral diseases separately, due to our retrospective study setting not allowing this. Moreover, two of the patients did not undergo a clinical or radiological examination because they were edentulous, which increases the risk for error in our analyses. However, we chose to include these patients, since the examinations would not have changed their MTDI scores. Another limitation is the relatively small sample size. However, we included all eligible patients in Finland, and this country-wide setting is a strength of our study. Despite its limitations, our study provides novel results on the possible connection between poor oral health and post-LT acute rejection, and the results remain indicative despite these limitations. However, despite the link, common confounders might affect both poor oral health and acute rejection post-LT. Therefore, further studies examining confounders affecting this relationship are needed. A possible area of further research based on this study would be the impact of oral diseases on post-LT complications, especially acute rejection. Another interesting topic for further study would be to assess the impact of receiving periodontal treatment prior to transplantation, while adjusting for the stage of periodontal disease.

### Conclusion

In conclusion, poor pre-LT oral health seems to be associated with an increased risk of post-LT acute rejection. Poor oral health is also associated with elevated ALT levels and decreased albumin levels, suggesting an effect on post-LT liver health. Therefore, attention should be given to treating oral and dental issues early in the course of liver disease and to highlighting the importance of maintaining good oral hygiene.

## Data Availability

The raw data supporting the conclusion of this article will be made available by the authors, without undue reservation.
