# Supplementary material for: Association Between Pre-Transplant Oral Health and Post-Liver Transplant Complications
Source: Transpl Int. 2023 Sep 12;36:11534. doi: 10.3389/ti.2023.11534 (PMC10520246; doi:10.3389/ti.2023.11534)
Supplement: Supplementary file 1 [file Table1.DOCX]

| **Supplementary table 1.** Basic characteristics of the LT patients and the underlying indication for transplantation. | | | |
| --- | --- | --- | --- |
| Parameter | Patients with dental data | Patients without dental data | p-value |
| Number of patients | 225 | 40 |  |
| Number of liver transplantations | 233 | 40 |  |
| Age^a^ (years) | 48.6 (±12.2) | 45.5 (±13.6) | 0.137 |
| Sex^b^ (male/female) | 127 (56.4)/98 (43.6) | 11 (28%)/29 (73%) | **<0.001** |
| Indication for transplantation^b^ |  |  |  |
| *Chronic liver disease* | 177 (79%) | 6 (15%) |  |
| *I) Primary sclerosing cholangitis* | *50 (22%)* | *2 (5%)* |  |
| *II) Primary biliary cholangitis* | *31 (14%)* | *0 (0%)* |  |
| *III) Alcohol cirrhosis* | *43 (19%)* | *0 (0%)* |  |
| *IV) Cryptogenic cirrhosis/ NASH* | *23 (10%)* | *3 (8%)* |  |
| *V) Other cirrhosis* | *29 (12%)* | *0 (0%)* |  |
| *VI) Other CLD^c^* | *21 (9%)* | *1 (3%)* |  |
| *Acute liver failure* | 24 (11%) | 33 (83%) |  |
| *Tumor (all)^d^* | 23 (10%) | 0 (0%) |  |
| *I) Tumor (no other CLD)^e^* | *6 (3%)* | *0 (0%)* |  |
| Metabolic disease | 1(0.4%) | 1 (3%) |  |
| Complication data^b^ |  |  |  |
| *No of LTs: ≥1 complication* | 230 (99%) | 40 (100%) | 0.849 |
| *No. of LTs: no complications* | 3 (1%) | 0 (0%) |  |
| *No. of patients: Survival* |  |  | 0.897 |
| *I) Retransplantation* | 20 (9%) | 3 (8%) |  |
| *II) Death* | 70 (31%) | 11 (28%) |  |
| *No. of patients: Infection* | 154 (68%) | 28 (70%) | 1.000 |
| *No. of patients: cardiovascular disease* | 29 (13%) | 7 (18%) | 0.453 |
| *No. of patients: Incident diabetes* | 63 (28%) | 9 (23%) | 0.565 |
| *No. of patients: Hypertension* | 132 (59%) | 25 (63%) | 0.728 |
| *No. of patients: Cancer* | 61 (3%) | 7 (18%) | 0.262 |
| *No. of patients: Acute rejection* | 121 (54%) | 17 (43%) | 0.230 |
| *No. of patiets: Chronic rejection* | 5 (2%) | 1 (3%) | 1.000 |
| Dental parameters^a^ |  | - |  |
| *Mean MTDI score ^f^* | 2.6 (±1.9) |  |  |
| *DMFT score^g^* | 22.8 (±8.1) |  |  |
| *Number of teeth pre dental treatment^h^* | 23.2 (±8.5) |  |  |
| *Number of extracted teeth pre-LT^i^* | 3.2 (±4.3) |  |  |
| MELD score at LT^a^ | 18.6 (±8.3) | **-** |  |
| Laboratory values at 1-year post-LT^a^ |  | **-** |  |
| *P-ALT (U/l)* | 38 (±39) |  |  |
| *P-ALP (U/l)* | 150 (±115) |  |  |
| *P-Bilirubin (*µmol/l) | 15 (±9) |  |  |
| *P-Albumin (g/l)* | 38 (±4) |  |  |
| *P-GGT (U/l)* | 98 (±185) |  |  |
| *P-CRP (mg/l)* | 4 (±14) |  |  |
| Abbreviations: ALP = alkaline phosphatase, ALT = alanine aminotransferase, CLD = chronic liver disease, CRP = c-reactive protein, DMFT = decayed, missing, filled teeth, GGT = gamma-glutamyl transferase, LT = liver transplantation, MELD = model of end-stage liver disease, MTDI = modified total dental index, NASH = non-alcoholic steatohepatitis  ^a^Data given as mean (SD)  ^b^Data given as n (%)  ^c^Other chronic liver diseases includes Budd–Chiari disease, polycystic disease, extrahepatic biliary atresia, congenital biliary fibrosis, alpha-1 antitrypsin deficiency, choledochal cyst, Caroli disease, and cystic fibrosis  ^d^All liver transplantations with a tumor as the underlaying cause  ^e^Liver transplantations with a tumor as the only underlaying cause  ^f^2 patients missing data  ^g^15 patients missing data  ^h^5 patients missing data  ^i^3 patients missing data  p-values for nonparametric variables were analyzed using Mann–Whitney U test. p-values for group variables were analyzed using Fisher’s exact test. | | | |
