# Supplementary figures and images for: Association Between Pre-Transplant Oral Health and Post-Liver Transplant Complications
Source: Transpl Int. 2023 Sep 12;36:11534. doi: 10.3389/ti.2023.11534 (PMC10520246; doi:10.3389/ti.2023.11534)

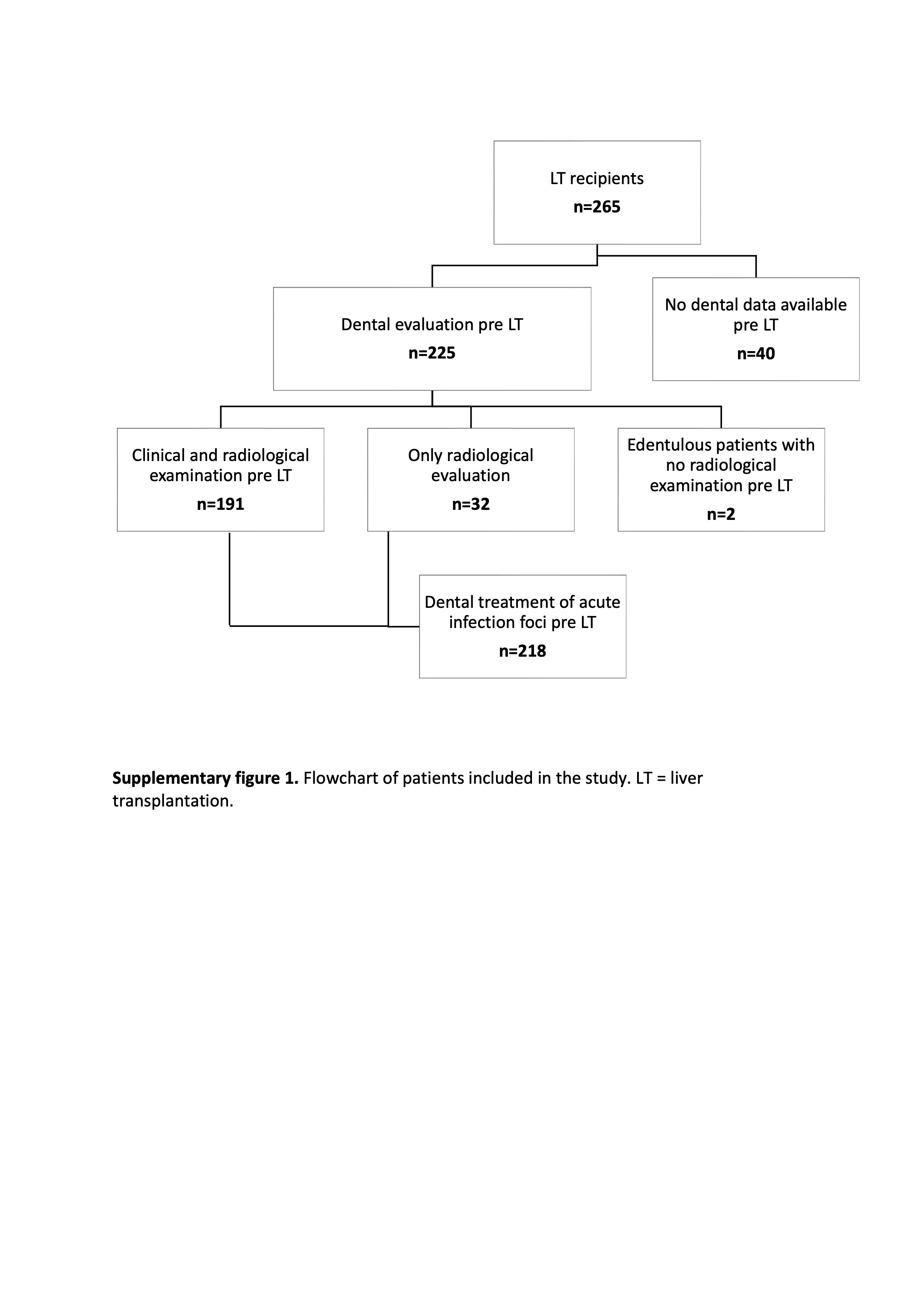

Supplement: Supplementary file 2 [file Image1.JPEG]
