# Supplementary material for: Association Between Pre-Transplant Oral Health and Post-Liver Transplant Complications
Source: Transpl Int. 2023 Sep 12;36:11534. doi: 10.3389/ti.2023.11534 (PMC10520246; doi:10.3389/ti.2023.11534)
Supplement: Supplementary file 3 [file Table2.DOCX]

| **Supplementary table 2.** Univariate Cox regression analysis of the correlation between pre-LT dental status based on MTDI groups (low (0–2), medium (3), high (4–10)) and post-LT complications. Follow-up time until end of July 2020. | | | | |
| --- | --- | --- | --- | --- |
| **Complication** | **Number of events** | **Hazard ratio** | **CI (95%)** | **p-value** |
| Survival^a^ | 89 |  |  |  |
| *Low MTDI* |  | Reference |  |  |
| *Medium MTDI* |  | 1.15 | 0.66–2.00 | 0.620 |
| *High MTDI* |  | 1.37 | 0.85–2.20 | 0.199 |
| Infection^b^ | 147 |  |  |  |
| *Low MTDI* |  | Reference |  |  |
| *Medium MTDI* |  | 1.19 | 0.78–1.81 | 0.413 |
| *High MTDI* |  | 0.95 | 0.65–1.40 | 0.810 |
| Cardiovascular disease | 29 |  |  |  |
| *Low MTDI* |  | Reference |  |  |
| *Medium MTDI* |  | 1.84 | 0.71–4.75 | 0.208 |
| *High MTDI* |  | 2.17 | 0.94–5.00 | 0.070 |
| Incident diabetes | 61 |  |  |  |
| *Low MTDI* |  | Reference |  |  |
| *Medium MTDI* |  | 1.18 | 0.63–2.21 | 0.596 |
| *High MTDI* |  | 0.83 | 0.44­–1.55 | 0.557 |
| Hypertension | 127 |  |  |  |
| *Low MTDI* |  | Reference |  |  |
| *Medium MTDI* |  | 1.00 | 0.63–1.60 | 0.992 |
| *High MTDI* |  | 1.31 | 0.87–1.96 | 0.194 |
| Cancer | 59 |  |  |  |
| *Low MTDI* |  | Reference |  |  |
| *Medium MTDI* |  | 0.74 | 0.43–1.80 | 0.735 |
| *High MTDI* |  | 1.28 | 0.72–2.30 | 0.399 |
| Acute rejection | 115 |  |  |  |
| *Low MTDI* |  | Reference |  |  |
| *Medium MTDI* |  | 1.28 | 0.78–2.08 | 0.331 |
| *High MTDI* |  | 1.70 | 1.13–2.58 | **0.012** |
| Chronic rejection | 4 |  |  |  |
| *Low MTDI* |  | Reference |  |  |
| *Medium MTDI* |  | 0.00 | 0.00 | 0.980 |
| *High MTDI* |  | 0.67 | 0.07–6.48 | 0.733 |

Abbreviations: LT = liver transplantation, MTDI = modified total dental index

Only patients whose teeth were treated pre-LT were included in the analyses (n = 218)

^a^Transplant survival time in months from transplantation until death, re-transplantation, or end of follow-up (31.7.2020)

^b^Time from transplantation until first infection episode
